# Near-Fatal, Delayed Diagnosis of Kingella kingae Endocarditis in an Infant

**DOI:** 10.7759/cureus.92229

**Published:** 2025-09-13

**Authors:** Mathias T Clausen, Shahin Gaini, Jan Joanesarson, Jesper N Steensberg, David Kocemba

**Affiliations:** 1 Department of Internal Medicine, National Hospital of the Faroe Islands, Tórshavn, FRO; 2 Department of Pediatrics and Adolescent Medicine, Rigshospitalet, Copenhagen, DNK; 3 Department of Orthopaedics, Horsens Hospital, Horsens, DNK

**Keywords:** cardiac arrest, cerebral infarction, hacek, infectious disease, infective endocarditis, kingella kingae, pediatric infection

## Abstract

We report a case of a 15-month-old male patient who presented with a two-week history of fever, lethargy, and mild respiratory symptoms. Initial investigations showed markedly elevated inflammatory markers, and chest imaging was interpreted as pneumonia. The patient was discharged on oral amoxicillin. Blood cultures later identified *Kingella kingae *(*K. kingae*), but no change in antibiotic therapy or follow-up was initiated due to apparent clinical improvement. Thirteen days later, the patient was readmitted in septic condition with a large mitral valve vegetation. He developed cardiac arrest during anesthesia induction and underwent emergent mitral valve reconstruction. Postoperative neuroimaging revealed multiple infarctions in the left middle cerebral artery territory. The patient is recovering with right-sided hemiparesis and continues to receive multidisciplinary rehabilitation and cardiologic follow-up. This case underscores the importance of considering infective endocarditis in pediatric patients with *K. kingae* bacteremia, a rare organism with limited literature on its clinical spectrum, even in the absence of classic features. Early echocardiographic assessment is critical to reduce the risk of severe complications.

## Introduction

*Kingella kingae* (*K. kingae*) is an emerging pediatric pathogen known to cause invasive infections, most commonly osteomyelitis and septic arthritis [1]. Although less frequent, *K. kingae* can also lead to more serious conditions such as pneumonia and infective endocarditis [1].

Infective endocarditis caused by *K. kingae* is exceptionally rare and often severe, with only 46 cases described throughout the literature [1, 2]. Approximately 75% of patients with invasive *K. kingae* disease are younger than 18 months; however, a mean age of 25 months was reported in a small Israeli cohort with *K. kingae* endocarditis [3,4]. Serious complications may include embolic stroke, meningitis, brain abscess, heart failure, and cardiogenic shock [4]. While the overall pediatric mortality rate from infective endocarditis ranges between 5% and 10%, it exceeds 10% in confirmed *K. kingae* endocarditis cases [5]. Delayed identification of the organism has been suggested as a key factor contributing to disease severity [4].

Therefore, recognizing, diagnosing, and initiating treatment at the earliest is indispensable to improving clinical outcomes. Appropriate antimicrobial therapies are critical to reducing morbidity and mortality. We describe a case of delayed diagnosis of *K. kingae* endocarditis in a 15-month-old male patient, which progressed to severe cardiac and neurologic complications, requiring emergent mitral valve surgery and resulting in postoperative cerebral infarctions. This case is reported in accordance with the CAse REport (CARE) guidelines for case reports [6].

## Case presentation

Patient information

A 15-month-old Caucasian male with no significant past medical history was admitted to the pediatric unit at the National Hospital of the Faroe Islands in Tórshavn, Faroe Islands (Table 1).

**Table 1:** Timeline: symptoms, events, findings, and treatment GP: general practitioner; CRP: C-reactive protein; CXR: chest X-ray; MALDI-TOF: matrix-assisted laser desorption ionization time-of-flight; TTE: transthoracic echocardiography; HR: heart rate; RR: respiratory rate; CT: computed tomography; MRI: magnetic resonance imaging; MCA: middle cerebral artery

Date	Event/Intervention	Findings/Outcome
Day -14 to 0	Initial symptoms develop; GP consultation and three nurse calls.	Fever, fatigue, mucus regurgitation, and mild respiratory distress. Viral illness suspected.
Day 0	Admission to The National Hospital of the Faroe Islands	CRP 237 mg/L, leukocytosis, pulmonary crackles, CXR: suspected perihilar pneumonia, blood cultures drawn
Day 1	Microscopy of blood culture	Gram-negative rods detected; patient discharged with presumedcommunity-acquired pneumonia
Day 2–13	Outpatient period	Culture identified *Kingella kingae* via MALDI-TOF; no treatment change or follow-up
Day 14	Readmission	Lethargy, anorexia, dehydration; new murmur; CRP 6 mg/L, leukocytes normal; TTE: mitral valve vegetation (1.1 × 1.7 cm)
Day 15	Transfer to a tertiary care center (Rigshospitalet, Copenhagen) and surgery	Patient arrived septic; HR 180–190, RR up to 70; planned for mitral valve surgery. Cardiac arrest during induction, resuscitation successful; mitral valve reconstructed
Day 20	Neurologic deterioration	CT- and MRI-scan: infarction in left MCA territory and thalamic lacunar infarcts
Week 1–7 (post-op)	Inpatient recovery and rehabilitation	Ceftriaxone × six weeks, ciprofloxacin × two weeks; neurorehabilitation; discharged on warfarin and acetylsalicylic acid
Month 20	Re-operation	Mitral valve replacement due to progressive stenosis; uncomplicated
Month 20+	Ongoing follow-up	Biannual follow-up

The caregivers reported a two-week history of fluctuating fever, fatigue, mild respiratory distress, and nightly episodes of mucus regurgitation. Prior to admission, the patient was evaluated once in person by a general practitioner and had three telephone consultations with a nurse, each initiated by the caregivers due to persistent fever and lethargy. A minor viral illness was suspected during each encounter. The child attended a daycare facility and had no relevant family or psychosocial history.

Clinical findings

**Figure 1:**
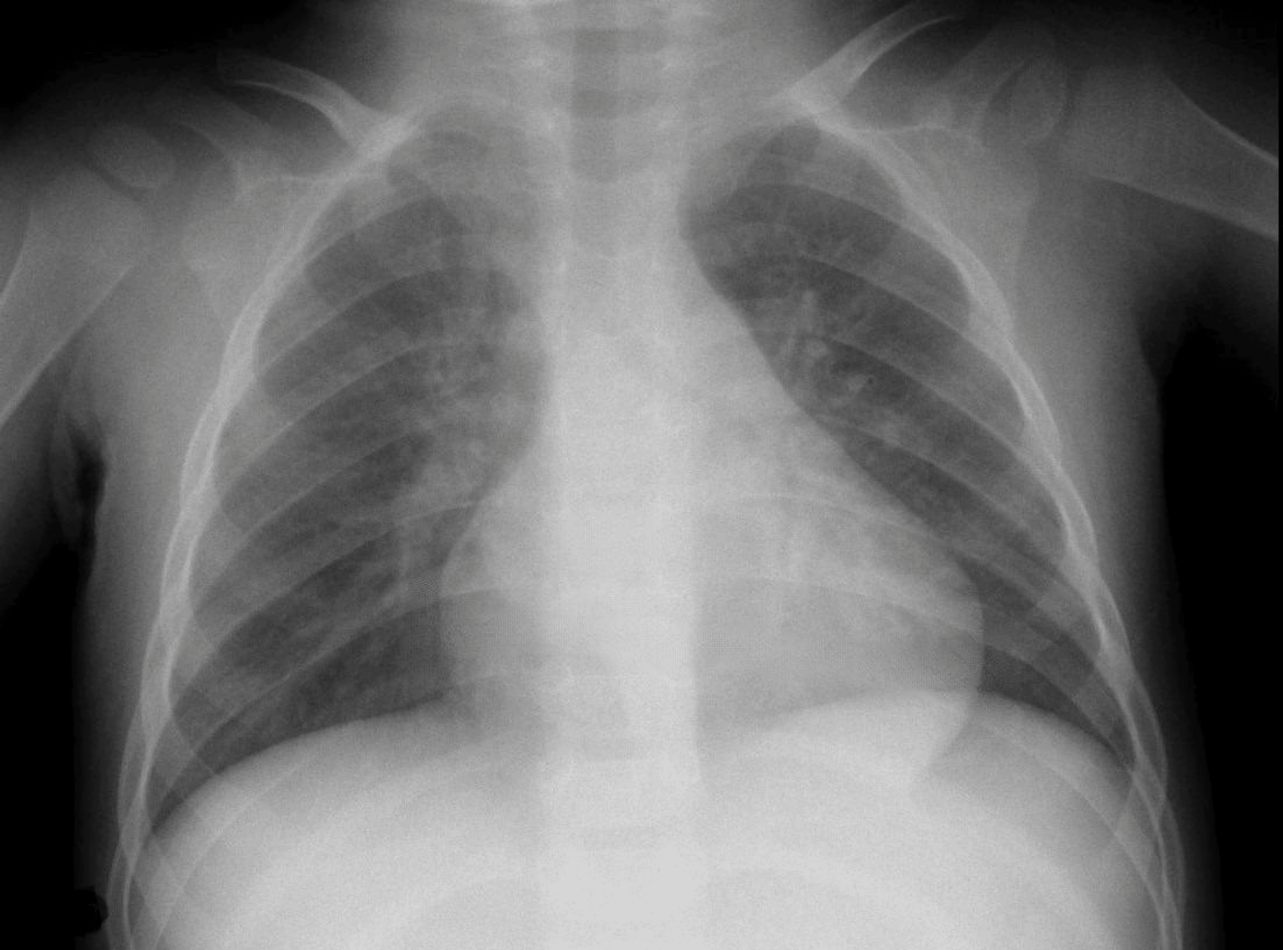
Chest X-ray (anterior-posterior view)

On admission, the patient appeared calm, with no signs of respiratory distress or cyanosis. Vital signs included a heart rate of 160 beats per minute, oxygen saturation of 99%, and a body temperature of 37.8°C. Pulmonary auscultation revealed bilateral crackles, while cardiac auscultation demonstrated a normal sinus rhythm without murmur. Laboratory evaluation showed leukocytosis (24.2 × 10⁹/L) with neutrophil predominance (17.4 × 10⁹/L) and markedly elevated C-reactive protein (CRP) at 237 mg/L. Venous blood gas values were within normal limits. Urinalysis (dipstick) was negative for signs of urinary tract infection. Blood cultures were obtained at admission. Chest X-ray (CXR) was interpreted by the admitting pediatrician as showing possible right-sided perihilar pneumonia (Figure 1).

Diagnostic assessment

On the day following admission, microscopy of the blood cultures (BACTEC™ Blood Culture System, Becton, Dickinson and Company, NJ, USA) revealed gram-negative rods. A radiologist subsequently re-evaluated the CXR and found it to be normal, contradicting the initial interpretation of pneumonia. Despite these findings, the patient showed clinical improvement and was discharged with a diagnosis of bacterial pneumonia.

After discharge, the blood culture isolate was identified as *K. kingae* using matrix-assisted laser desorption/ionization time-of-flight mass spectrometry (MALDI-TOF; Bruker MALDI-TOF, Bruker Corporation, Billerica, MA). The isolate was detected in one out of one blood culture bottle. The bacterial isolate did not grow on subculture; therefore, susceptibility testing was unsuccessful. No change was made to the patient’s empirical antibiotic regimen, and no follow-up or recall was initiated.

**Figure 2:**
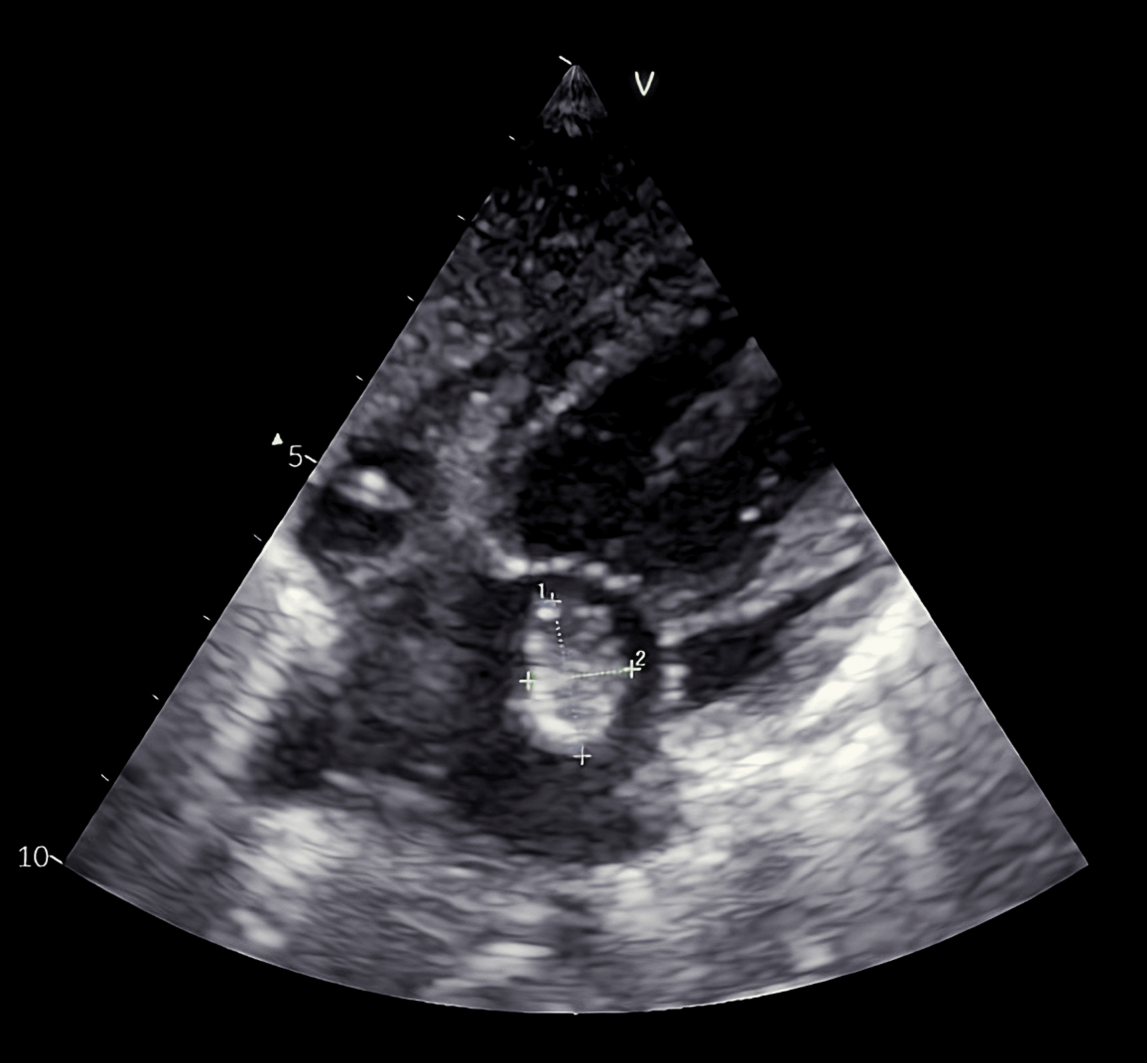
Subcostal view of the transthoracic echocardiography v: left

Thirteen days later, the patient was readmitted due to clinical deterioration. He was lethargic, anorectic, and dehydrated. Vital signs included a heart rate of 170 beats per minute and a temperature of 37.4°C. Cardiac auscultation revealed a new holosystolic murmur. Laboratory evaluation showed a low CRP (6 mg/L) and a normal leukocyte count. Transthoracic echocardiography (TTE) revealed a mobile mitral valve vegetation measuring 1.1 × 1.7 cm (Figure 2 and Video 1).

**Video 1:** Parasternal long axis view (PLAX) of the transthoracic echocardiography v: left

Upon arrival at Rigshospitalet in Copenhagen, Denmark, the patient appeared critically ill and septic, with a heart rate of 180-190 beats per minute and a respiratory rate of up to 70 breaths per minute.

Therapeutic intervention

At initial presentation, the patient was treated empirically for presumed community-acquired pneumonia with oral amoxicillin at 60 mg/kg/day for seven days. Following identification of mitral valve vegetation on transthoracic echocardiography during readmission, antimicrobial therapy was escalated to intravenous (IV) ampicillin (200 mg/kg/day), IV cloxacillin (30 mg/kg/day), and a single dose of IV gentamicin (50 mg).

Due to the severity of the findings and the urgent need for surgical management, the patient was transferred by air ambulance to Rigshospitalet in Copenhagen. Emergent mitral valve reconstruction was planned. During anesthesia induction, the patient experienced cardiac arrest, requiring 10 minutes of cardiopulmonary resuscitation. Surgery proceeded with reconstruction of the mitral valve using an equine pericardial “Heimlich” sleeve valve.

Postoperatively, antimicrobial therapy was adjusted based on blood culture findings. The patient received IV ceftriaxone (100 mg/kg once daily) for six weeks and IV ciprofloxacin (20 mg/kg three times daily) for two weeks. Ciprofloxacin was continued until cerebral imaging excluded the presence of a brain abscess.

Anticoagulation was initiated with warfarin for three months following surgery. The patient was then transitioned to lifelong antiplatelet therapy with oral acetylsalicylic acid (37.5 mg daily).

Rehabilitation included inpatient and outpatient multidisciplinary neurorehabilitation comprising physiotherapy, occupational therapy, speech therapy, and neuropsychiatric care. The patient remained hospitalized for seven weeks after transfer and was discharged following stabilization of both cardiac and neurologic status. All treatments were well tolerated, and no adverse drug reactions were reported.

Follow-up and outcomes

Following surgery, the patient demonstrated right-sided hemiparesis, accompanied by delayed motor development, cognitive deficits, and impaired speech, when compared to age-matched peers. He underwent multidisciplinary neurorehabilitation, including physiotherapy, occupational therapy, speech therapy, and neuropsychiatric evaluation. Subsequent assessment by a pediatric neuropsychiatrist indicated cognitive abilities consistent with age norms.

Due to progressive mitral stenosis, a second cardiac surgery was performed 20 months after the initial operation. The prior equine pericardial sleeve valve was replaced with a new biological prosthesis of the same material. The procedure was uneventful.

The patient continues to receive biannual follow-up in the pediatric cardiology outpatient clinic, including transthoracic echocardiography and developmental assessments. He remains on long-term prophylaxis with acetylsalicylic acid (37.5 mg daily) and had received warfarin for the first three postoperative months. At more than 30 months of follow-up, the patient shows significant functional recovery.

## Discussion

*K. kingae *is a gram-negative coccobacillus that commonly causes osteoarticular infections and bacteremia in children aged between six and 36 months. Endocarditis is a rare manifestation. The high prevalence in young children is thought to be associated with immature humoral immunity and low antibody titers, particularly in daycare environments where close contact facilitates transmission [4].

*K. kingae* primarily colonizes the oropharynx, with reported asymptomatic carriage rates ranging from 3% to 17% in children [7]. Most colonized individuals remain asymptomatic, and only a minority develop invasive disease [8]. Certain virulent strains have been identified, with molecular studies demonstrating matching clones at both the oropharynx and the site of infection [9]. Contributing factors such as viral co-infection and the expression of an RTX cytotoxin are believed to facilitate mucosal invasion, bloodstream dissemination, and seeding of distant sites [7, 9, 10].

*K. kingae* is transmitted primarily via respiratory droplets and close contact [11]. Outbreaks of invasive disease have been documented in daycare settings, where antibiotic prophylaxis was occasionally administered to exposed children [12-14]. Although our patient attended a daycare facility, no other cases of invasive *K. kingae* infection were reported during the same period.

*K. kingae* belongs to the HACEK group (which includes *Haemophilus*, *Aggregatibacter*, *Cardiobacterium*, *Eikenella*, and *Kingella *species) of fastidious gram-negative bacteria, which account for only 1.4% to 3% of infective endocarditis cases in adults [15]. Pediatric endocarditis caused by *K. kingae* is exceedingly rare. The number of published reports has increased since 1990, likely due to advances in diagnostic technology [16]. Improved identification methods include the inoculation of specimens into enriched blood culture media and the use of sensitive nucleic acid amplification assays, which have enabled detection in previously culture-negative infections.

In a nationwide Israeli cohort, endocarditis accounted for only 2.5% of pediatric invasive *K. kingae* infections, with the majority presenting as osteoarticular infections (52.6%) or occult bacteremia (43.6%) [3]. Certain clonal strains of *K. kingae *have been associated with specific clinical syndromes, although asymptomatic carriage of these virulent clones is also well documented [17]. While most invasive *K. kingae* infections follow a benign course, endocarditis is a notable exception and may lead to life-threatening complications [4].

Reported complications of *K. kingae* endocarditis include embolic stroke, cardiac valve rupture, meningitis, brain abscess, cardiogenic shock, and death [4, 18]. In our case, the disease course was marked by prolonged fever (≥26 days), delayed microbiological follow-up after positive blood cultures, and eventual cardiac and neurologic deterioration. The cardiac arrest during anesthesia induction was likely multifactorial, involving both septic and cardiogenic shock. Cerebral infarctions identified postoperatively may have resulted from septic embolism, intraoperative hypoperfusion, or coagulopathy. No brain abscesses were observed.

## Conclusions

This case illustrates the potential severity of *K. kingae* endocarditis in young children and the consequences of delayed diagnosis. Although typically associated with milder infections, *K. kingae* can cause fulminant endocarditis with life-threatening cardiac and neurologic complications. When *K. kingae* is identified in blood cultures, particularly in febrile children with prolonged or unexplained symptoms, infective endocarditis must be promptly excluded using echocardiography. Early recognition and appropriate intervention are essential to improving outcomes in this rare but serious disease.
