# Correction: Near-Fatal, Delayed Diagnosis of Kingella kingae Endocarditis in an Infant

**DOI:** 10.7759/cureus.c324

**Published:** 2025-09-25

**Authors:** Mathias T Clausen, Shahin Gaini, Jan Joanesarson, Jesper N Steensberg, David Kocemba

**Affiliations:** 1 Department of Internal Medicine, National Hospital of the Faroe Islands, Tórshavn, FRO; 2 Department of Pediatrics and Adolescent Medicine, Rigshospitalet, Copenhagen, DNK; 3 Department of Orthopaedics, Horsens Hospital, Horsens, DNK

This article has been revised to change the affiliation for David Kocemba. His affiliation has been changed from "Department of Clinical Medicine, Aarhus University, Aarhus, DNK" to "Department of Orthopaedics, Horsens Hospital, Horsens, DNK".

